# Advancement on Sustained Antiviral Ocular Drug Delivery for Herpes Simplex Virus Keratitis: Recent Update on Potential Investigation

**DOI:** 10.3390/pharmaceutics13010001

**Published:** 2020-12-22

**Authors:** Manisha Pandey, Hira Choudhury, Azila Abdul-Aziz, Subrat Kumar Bhattamisra, Bapi Gorain, Jocelyn Sziou Ting Su, Choo Leey Tan, Woon Yee Chin, Khar Yee Yip

**Affiliations:** 1 Department of Pharmaceutical Technology, School of Pharmacy, International Medical University, Bukit Jalil, Kuala Lumpur 57000, Malaysia; 2 Centre for Bioactive Molecules and Drug Delivery, Institute for Research, Development and Innovation, International Medical University, Kuala Lumpur 57000, Malaysia; 3 Department of Chemical and Environmental Engineering, Malaysia-Japan International Institute of Technology, Universiti Teknologi Malaysia, Jalan Sultan Yahya Petra, Kuala Lumpur 54100, Malaysia; r-azila@utm.my or; 4 Department of Life Sciences, School of Pharmacy, International Medical University, Bukit Jalil, Kuala Lumpur 57000, Malaysia; subratkumar@imu.edu.my; 5 School of Pharmacy, Faculty of Health and Medical Sciences, Taylor’s University, Subang Jaya, Selangor 47500, Malaysia; bapi.gorain@taylors.edu.my; 6 Center for Drug Delivery and Molecular Pharmacology, Faculty of Health and Medical Sciences, Taylor’s University, Subang Jaya, Selangor 47500, Malaysia; 7 Undergraduate, School of Pharmacy, International Medical University, Bukit Jalil, Kuala Lumpur 57000, Malaysia; JOCELYN.SU@student.imu.edu.my (J.S.T.S.); TAN.CHOOLEEY@student.imu.edu.my (C.L.T.); CHIN.WOONYEE@student.imu.edu.my (W.Y.C.); YIP.KHARYEE@student.imu.edu.my (K.Y.Y.)

**Keywords:** ocular drug delivery, herpes simplex virus keratitis, mucoadhesive, in situ ophthalmic gel, novel approaches, safety

## Abstract

The eyes are the window to the world and the key to communication, but they are vulnerable to multitudes of ailments. More serious than is thought, corneal infection by herpes simplex viruses (HSVs) is a prevalent yet silent cause of blindness in both the paediatric and adult population, especially if immunodeficient. Globally, there are 1.5 million new cases and forty thousand visual impairment cases reported yearly. The Herpetic Eye Disease Study recommends topical antiviral as the front-line therapy for HSV keratitis. Ironically, topical eye solutions undergo rapid nasolacrimal clearance, which necessitates oral drugs but there is a catch of systemic toxicity. The hurdle of antiviral penetration to reach an effective concentration is further complicated by drugs’ poor permeability and complex layers of ocular barriers. In this current review, novel delivery approaches for ocular herpetic infection, including nanocarriers, prodrugs, and peptides are widely investigated, with special focus on advantages, challenges, and recent updates on in situ gelling systems of ocular HSV infections. In general congruence, the novel drug delivery systems play a vital role in prolonging the ocular drug residence time to achieve controlled release of therapeutic agents at the application site, thus allowing superior ocular bioavailability yet fewer systemic side effects. Moreover, in situ gel functions synergistically with nanocarriers, prodrugs, and peptides. The findings support that novel drug delivery systems have potential in ophthalmic drug delivery of antiviral agents, and improve patient convenience when prolonged and chronic topical ocular deliveries are intended.

## 1. Introduction

**Figure 1:**
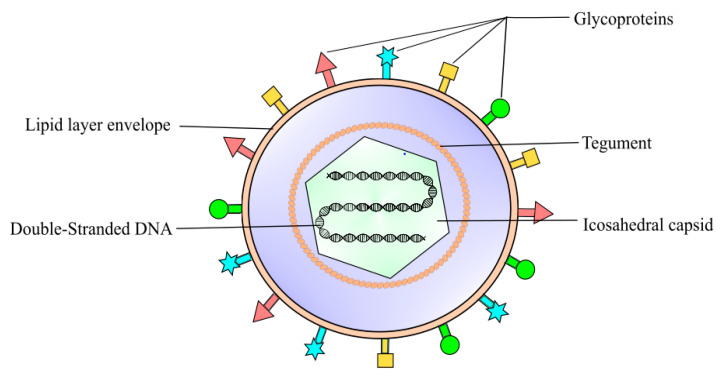
Herpes simplex virus 1 (HSV-1) is classified into the Alphaherpesvirinae family, a sub-family of Herpesviridae. It is an enveloped DNA virus consisting of a linear double-strand genome protected inside an icosahedral capsid. The inner tegument is made up of a layer of mRNA and proteins, whereas the outer lipid bilayer envelope contains glycoproteins. These glycoproteins are responsible for the invasion of the virus into the host cell.

Eye infection is a prevalent problem in primary care and remains a crucial healthcare concern. According to the American Academy of Ophthalmology (AAO), herpes simplex virus (HSV) keratitis (HSK) is the leading cause of infectious blindness worldwide [1]. HSK is defined as a corneal inflammatory condition caused by the HSV infection [1,2]. The global incidence of herpetic keratitis is estimated at 1.5 million per year, resulting in 40,000 new cases of severe visual impairment associated with corneal scarring and opacification [3,4]. HSV type I (HSV-1) is by far the most predominant causative pathogen of eye infections (Figure 1) [1]. HSV-1 is also known for causing orolabial herpes, HSV folliculitis, herpes gladiatorum, herpetic whitlow, and eczema herpeticum [4,5,6]. HSV can be transferred to the eye by touching an active lesion and then the eye [1]. The National Health and Nutrition Evaluation revealed a seroprevalence of HSV-1 in 53.9% of 14–49 year olds, and 90% of adults 50 years or older [7,8], indicating that the majority of the population has been exposed to this virus thus are at risk of developing HSK.

The HSK manifestations can be categorised according to epithelial keratitis, stromal keratitis, and endotheliitis [1,5]. Most of the initial infection cases (80%) usually manifest as acute epithelial keratitis [2,9]. Patients may report symptoms like sudden eye pain and redness, watery discharge, photophobia, and blurry vision [1,2]. Under slit-lamp microbioscopy examination, the patterns of epithelial lesions can be differentiated into punctate, dendritic, or geographic ulcers [1,5]. Punctate lesions appear like granular vesicles which can rapidly coalesce into a linear branching dendritic pattern [1,10]. Further dendritic extensions at the edges can cause geographic ulcers [1]. These lesions contain highly contagious and active replicating viruses, which results in the desquamation of the corneal structure. Without appropriate and prompt treatment, patients may suffer prolonged infection, making them susceptible to recurrent keratitis [9,10,11]. Although the symptoms typically affect the unilateral eye, bilateral infection is common in younger age groups and immunocompromised patients [12]. Compared to adults, children have overall worse visual outcomes and hence they are at greater risk of permanent vision loss from amblyopia [12]. Furthermore, the impact of the disease in developing nations with limited access to treatment and immunosuppression perhaps contributes to a significantly higher visual morbidity than currently known [3,12].

The Herpes Eye Disease Study (HEDS) recommends topical formulations containing antiviral agents as the front-line treatment for herpes epithelial keratitis [1]. Undeniably, topical formulations are the most popular choices for ocular treatment as they are convenient to use. Nonetheless, there are multiple drawbacks associated with the present topical antiviral agents. Limited ocular bioavailability and a lack of controlled drug release profile are factors that hinder the effectiveness of topical antiviral formulations. Oral antiviral drugs may be the alternative treatment, but when given at high doses, the drugs can cause untoward systemic toxicities [13]. Hence, the need the leverage pharmaceutical technology to fabricate a more efficient, sustained release, and affordable antiviral topical formulations to halt disease progression and further elucidate their prospective roles in HSK prevention. This review article aims to investigate the potential of novel approaches in improving HSK topical treatment, including mucoadhesive systems, nanocarriers, prodrugs, peptide drugs, and in situ ocular formulations. The advantages, recent updates, and challenges of thermo-responsive, pH-responsive, ion-responsive, and multi-stimulus-responsive in situ gelling systems for various ocular diseases and HSK are extensively discussed. The ocular safety profile and clinical trials of the in situ gelling systems are included as well.

## 2. Pathophysiology of Herpes Simplex Virus Keratitis Condition

**Figure 2:**
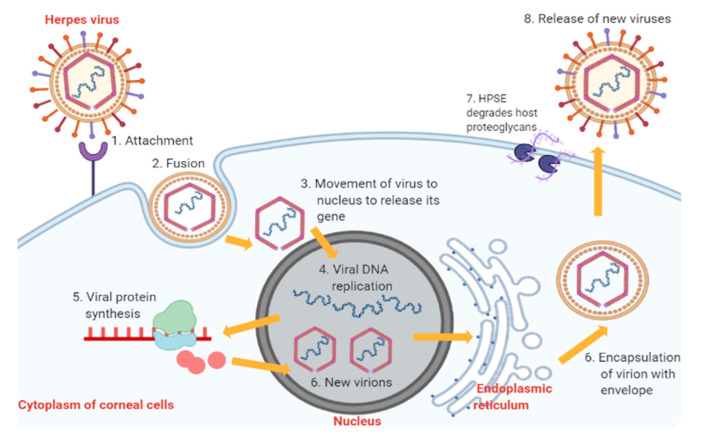
Stages of HSV-1 replication in corneal epithelial cells. Abbreviation: HPSE, heparanase, an enzyme which cleaves heparan sulfate proteoglycans to prevent virions from being trapped by the proteoglycans, thus promoting virion egress.

The pathophysiology of HSK involves complex sequential processes. Primary active infection begins with HSV-1 entry into corneal epithelial cells (Figure 2) [10]. The attachment of HSV-1 glycoproteins to the receptors on the epithelial cell surface enables the viral envelope to fuse with the cytoplasmic membrane [10,14]. After that, the viral tegument and nucleocapsid proteins are released into the host cytoplasm [14]. The capsid then moves towards the nucleus to begin viral DNA replication using host DNA polymerase while the virus mRNA is being transcribed and translated into new proteins [14]. The proteins and viral genes are assembled in the nucleus to form new virions, which travel to the endoplasmic reticulum and Golgi apparatus to acquire new envelopes [14]. In the end, HSV-1 results in cytolysis of infected epithelial cells while the new viral progenies are released with the help of heparanase (HPSE) and, subsequently, they infect other neighbouring cells [14].

Following acute epithelial infection, cytokines facilitate the infiltration of polymorphonuclear leucocytes, natural killer cells, macrophages, and Langerhans cells into the corneal stroma and endothelium layers [14,15]. Type I interferon (IFN-a/b), HSV-specific IgG, and IgA may help prevent virus spread [10]. However, uncontrolled viral multiplication may happen specifically among patients who are immunocompromised [10]. Subsequently, it may result in corneal basement membrane rupture and initiation of stromal disease [10]. As HSV is neurotropic, some may stay dormant in optic nerves or trigeminal nerves before reactivation [15]. Virus reactivation is likely to happen due to a combination of host, virus, and environmental factors, which can be stress, hormonal changes, ultraviolet light exposure, and laser treatment [10].

Stromal and endothelial keratitis results primarily from the host’s immune response towards the virus spread from epithelial infection or viral reactivation. It is postulated that the recruited pro-inflammatory cytokines and growth factors initially assist in virus removal but later cause tissue destruction, scarring, and neovascularisation [14]. Connective tissue in corneal scars is organised abnormally, leading to increased light scatter and corneal opacity [14]. Some other complications of HSK are neurotrophic keratopathy and superinfection [10]. Moreover, patients had reported significant impairment in quality of life associated with ocular pain, especially those who encounter multiple relapses [16]. Therefore, antiviral treatment is essential in suppressing antiviral spread to prevent the severe sequelae of HSK.

## 3. Available Treatments for Herpes Simplex Virus Keratitis and Associated Limitations

According to the AAO HSK Treatment Guideline 2014, the treatment approach varies depending on the classification and the severity of the ocular infection [1]. The goal of treatment is to minimise corneal scarring, delay progression of stromal damage and improve the patient’s quality of life [1]. In general, the therapeutic interventions for HSK include antiviral agents, immunosuppressive agents, debridement, and surgical transplantation. There are advantages and challenges associated with each treatment option. The AAO HSK Treatment Guideline 2014 and HEDS recommend topical antiviral agents as first-line pharmacotherapy for epithelial HSK. The early generation antivirals are idoxuridine, iododesoxycytidine, vidarabine, and trifluridine [1,9,14,17,18]. Except for trifluridine, the rest of them have been discontinued due to reported harmful ocular side effects. Trifluridine is a synthetic pyrimidine nucleoside that inhibits thymidine incorporation into replicating DNA, thereby preventing the production of functional viral proteins and new virions. Trifluridine 1% ophthalmic solution was the United States Food and Drug Administration (FDA)-approved treatment for treating epithelial HSK and keratoconjunctivitis. It is used up to nine times daily for one week; the dose is then tapered down after one week. Due to the non-selective inhibition of DNA synthesis in both virus-infected and uninfected cells, trifluridine can cause local toxicities with prolonged use, i.e., ulceration, dysplasia, and canalicular stenosis. Hence, it should not be continuously used for more than 21 days due to a high risk of ocular toxicity [1]. These ocular toxicities have led to the decline of trifluridine use compared to new topical antivirals.

Acyclovir and ganciclovir are the new generation of antiviral agents. Acyclovir is a purine nucleoside with a better antiviral selectivity compared to trifluridine, thus having fewer ocular side effects [14]. Acyclovir comes in the form of ointment due to its lipophilicity characteristic and it is formulated with a polyethylene glycol (PEG) base [9,14]. Acyclovir ointment is widely used in other countries outside of the U.S., such as in Europe and Australia [11,19]. It is prescribed to patients with epithelial HSK with three to five times daily administration for one to three weeks [1]. Furthermore, ganciclovir is a synthetic purine nucleoside shown to be as effective as acyclovir in achieving a cornea cure rate [14,20]. Ganciclovir was marketed as FDA-approved 0.15% gel in 2009. Ganciclovir provides a greater antiviral spectrum, including human herpes virus, varicella-zoster virus, cytomegalovirus, Epstein–Barr virus, and adenovirus [9,14]. The ganciclovir molecule is relatively more potent than acyclovir in causing rapid apoptosis of HSV-infected cells. Thus, it is effective at a lower concentration (0.15% gel) compared to acyclovir (3% ointment) [9,14,21]. It can be given to patients with epithelial HSK with five times daily administration until healing of the corneal ulcer; the dose frequency is then tapered down to three times daily for a week [1]. Under some circumstances, the oral antiviral therapy has been used in place of topical agents in treating epithelial HSK. For example, when the patient is more susceptible to ocular surface toxicity (due to pre-existing eye disease) and paediatric patients refractory to topical antiviral [1,18].

At the same time, oral antiviral agents are indicated to treat HSV stromal and endothelial keratitis [1]. The oral antiviral agent aims to decrease viral load and reduce the magnitude of the inflammatory response in conjunction with a topical corticosteroid. Examples of oral antiviral agents are acyclovir, valacyclovir, and famciclovir. Valacyclovir has good systemic bioavailability and safety profile compared to acyclovir [14]. Sozen et al. reported that oral valacyclovir was more efficient in terms of epithelial healing rate and lower photophobia score [22]. Famciclovir is a prodrug of penciclovir proven to have clinical benefits in treating herpes virus with even better bioavailability [1,17]. Oral antivirals are also recommended as a prophylaxis treatment in patients with frequent recurrence of HSK, or undergoing any excimer laser photokeratectomy procedure [9,14]. However, resistance to acyclovir has become more prevalent, especially among immunocompromised patients. Alvarez et al. explained that approximately 3.5–10% of immunocompromised patients and 1% of immunocompetent patients reported resistance [17]. A novel approach in real-time cell analysis (RTCA) with a rapid result test compared to the gold standard phenotypic plaque reduction assay (PRA) is now emerging in the clinical field to encourage the screening of resistance to antivirals and provide a better treatment to patients [23,24,25].

Meanwhile, a topical corticosteroid is used in combination with oral antiviral agents in managing stromal HSK and endotheliitis. As discussed in the pathophysiology section, stromal HSV and endothelitiitis are complicated by underlying immune reaction and viral antigens. Corticosteroids are able to inhibit cellular immune response, opacification, scarring, and neovascularisation. Available corticosteroids include prednisolone acetate 1% suspension, prednisolone sodium phosphate 1% suspension, fluorometholone 0.1% suspension, rimexolone 1% suspension, and difluprednate 0.05% emulsion [1]. HEDS I reported that the corticosteroid treatment group showed a greater reduction (68%) in stromal keratouveitis progression compared to a placebo group [7,21]. However, the judicious use and dose tapering of corticosteroids is warranted to prevent adverse effects such as exacerbation of infection, corneal thinning, and steroid-induced glaucoma and cataract [1,7]. Some studies suggest topical cyclosporine or topical tacrolimus as supplementary immunosuppressive agents but require further clinical validation [26]. Debridement and cryopreserved amniotic membrane (CAM) may aid in re-establishing epithelial healing [27,28]. Lastly, corneal transplant may be an option for patients with severe corneal scarring, yet corneal graft rejection is another treatment challenge [29]. At present, research on the vaccine and new therapeutic molecules against the latent virus is still underway, which may take a substantial amount of time before they can be used in clinical settings.

In brief, topical antivirals are essential first-line agents to treat epithelial HSK at the earliest onset to suppress viral replication, maintain latency, and prevent complications. In oral formulations, a small amount of the drug will reach the posterior chamber of the eye, limited by the retinal blood barrier [13,30]. Besides, both oral acyclovir and valacyclovir may cause nephrotoxicity in renal impairment patients [1,14,17]. However, there are several limitations with current topical antiviral agents. Due to a lack of optimal sustained drug release properties, all of them require multiple doses (five to nine times). Particularly, ophthalmic solution is the most rapidly eliminated through the nasolacrimal route [13,31,32]. Ointment can cause blurred vision and sticky, greasy, and gritty sensations after application, thus reducing patient compliance [9,13,14]. Pre-formed gel can be difficult to use due to a thicker consistency compared to eye drops. Moreover, none of the current topical antiviral agents supports drug permeation into deeper ocular layers. The possibility of using topical agents in HSK prophylaxis without causing resistance is an area worthy of investigation. Hence, the following discussions aim to give insights into the novel strategies that can surpass current treatment challenges of HSK epithelial keratitis. The ultimate goal of ocular formulations is to efficiently cross both static and dynamic barriers of the eye and reach the targeted infection site without excessive exposure of patients to systemic toxicities [30,31].

Therefore, the physical barriers and the problems associated with conventional ocular formulations are the major challenges in ophthalmic drug development. The mucoadhesive polymeric approaches have gained much importance in recent years as they offer several advantages which minimise the challenges. Moreover, the mucoadhesive delivery system enables drug release at the site of action, which avoids enzymatic degradation in the gastrointestinal tract as well as the first-pass effect [33]. This system also promotes local absorption due to the rich blood supply of the mucosa, provides a localised effect, and subsequently improves ocular bioavailability along with therapeutic efficacy [33,34,35]. The next section of the article emphasises different ocular deliveries under the umbrella of advanced delivery approaches towards overcoming the issues of ocular delivery and effective treatment.

## 4. Novel Approach for Ocular Drug Delivery against Herpes Simplex Keratitis

There is general agreement that transferring antiviral medication through the ocular route is a challenging task due to the unique structure of the eye and the physicochemical properties of antiviral drugs. In the current scientific panorama, many efforts have been focused on improving the ocular availability of antiviral drugs for the treatment of herpetic eye diseases. This section emphasises a number of novel strategies, including nanocarriers, prodrugs, peptide conjugation, and in situ gelling systems as smart delivery systems of antiviral medications for ocular herpes infections.

### 4.1. Lipid-Based Nanocarriers for Ocular Drug Delivery

The use of lipid-based nanocarriers in drug delivery via various routes of administration, including ophthalmic, nasal, and systemic routes, has been widely recognised [36,37,38]. Lipid carriers are generally biocompatible and biodegradable. Several prominent lipid carrier systems for anti-herpetic drugs, such as liposomes, niosomes, solid lipid nanoparticles (SLNs), nanostructured lipid carriers (NLCs), and nanoemulsions will be discussed in detail. 

#### 4.1.1. Liposomes and Niosomes for Ocular Drug Delivery

Liposomes have been proposed as one of the advanced ocular drug delivery strategies for anterior and posterior eye segments in recent decades. Basically, liposomes consist of single or multiple phospholipid bilayers arranged in a vesicular shape, surrounding an inner aqueous core [39]. Liposomes are unique in that they can simultaneously encapsulate both lipophilic and hydrophilic drugs within the lipid bilayers and aqueous core, respectively [38,39]. Liposomes offer advantages over traditional ophthalmic formulations, not only because of their nanosize but also ability to interact with the corneal surface, thereby improving ocular drug penetration and ocular bioavailability [13,38]. Therefore, liposomes are appropriate as drug carriers of acyclovir and ganciclovir which have poor permeability. In relation to this context, Chetoni and colleagues investigated the pharmacokinetics of acyclovir liposomes (LIPO-ACVs) in the aqueous humour after being administered topically [40]. Phosphatidyl choline cholesterol and stearylamine were used to synthesise the positively charged, unilamellar liposomes in which acyclovir was entrapped [40]. This study found that LIPO-ACVs resulted in an 11-fold greater drug availability in the aqueous humour in comparison with the reference ointment. This exceptional ocular bioavailability of LIPO-ACVs was attributed to the ability of positively charged liposomes to interact with the negatively charged corneal surface, specifically the sialic acid residues of the corneal mucous layer [13,40]. Consequently, this interaction allowed an effective, higher drug release (50.25%) and penetration through the corneal layer, thereby sustaining the inhibitory concentration of acyclovir in the aqueous humour. In contrast, the drug release of ointment was lower (3.85%) and inconsistent. The reasons behind the ineffectual drug release from ointment could be due to inadequate contact of the ointment with the diffusion membrane, apart from the high affinity of acyclovir towards the petrolatum ointment vehicle, preventing drug release [40]. Despite improved drug corneal permeation, formulation improvement is necessary to achieve an effective inhibitory concentration for a longer duration, in comparison with the 120 min of current LIPO-ACVs, in order to minimise daily dosing frequency and overcome patient non-compliance. Similarly, ocular delivery of a poorly soluble drug with high efficacy is still a change for researchers. So, mucus-penetrating nanocarriers can be a potential option for enhancing therapeutic efficacy. In this context, Law et al. developed a liposome for acyclovir ocular delivery. The results of ex vivo transcorneal permeation showed higher corneal penetration of positively charged liposomes compared to a free drug suspension. Morphological observation showed complete coating of the corneal layer by positively charged liposomes, which enhanced the residence time and therapeutic efficacy [41].

In addition to enhanced ocular penetration, newer generation liposomes that undergo surface modifications have the potential to control and target drug delivery to a specific ocular tissue. By virtue of this, Asasutjarit et al. developed transferrin-conjugated liposomes for ganciclovir (Tf-GCV-LPs) directed at the virus-infected retina [42]. This study suggested that drug release was primarily dependent on diffusion. Particularly, transferrin could delay the diffusion of ganciclovir, causing slow and prolonged release over 12 h. Furthermore, the ganciclovir intracellular uptake studies were performed by HPLC and fluorescence methods. Both studies confirmed that the concentration of ganciclovir was significantly higher in APRE-19 cells incubated with the Tf-GCV-LPs, as compared to solution and another test formulation without transferrin. It could be deduced that transferrin promoted drug binding to the selective receptors on the cell surface, thereby enhancing the APRE-19 intracellular uptake of ganciclovir via endocyctosis [42]. In agreement with cellular uptake results, Tf-GCV-LP recorded the highest inhibitory activity against viral glycoprotein B expression when compared to other reference formulations. This was ascribed to the surface modification on liposomes, which confers effective cellular internalisation of Tf-GCV-LPs as well as protection against endosomes, allowing a controlled drug release to the cells [42]. Besides that, the in vitro cytotoxicity test based on an MTT assay concluded that the Tf-GCV-LPs did not cause toxicity to the ARPE-19 cells, with cell viability of 80–100% at various ganciclovir concentrations, except 200 ug/mL (maximum concentration). At the maximum concentration (200 ug/mL), ganciclovir was toxic to both virus-infected cells and rapidly dividing healthy cells like ARPE-19 cells, by causing an inappropriate environment for cell growth [42]. From this study, the Tf-GCV-LPs were proven effective at inhibiting viral replication in the posterior eye segment over a prolonged duration (12 h) compared to the study by Chetoni et al. [40] reported earlier (120 min). Thus, surface-modified liposomes are worthy of future investigations as a potential antiviral delivery to target deeper ocular tissues infected by viruses, such as herpes stromal keratitis and cytomegalovirus retinitis.

Apart from liposomal formulations, niosomes are another feasible lipid nanocarrier system for ocular drug delivery in the treatment of virus infection. Niosomes are slightly differ from liposomes, as niosomes are synthesised using non-ionic surfactants instead of phospholipids [13]. Moreover, niosomes possess better stability and longer shelf-life compared to liposomes [38]. Nevertheless, niosomes are equally permeable, biocompatible, and effective for sustained drug delivery of both hydrophilic and lipophilic drugs [13,38]. In view of this, Akhter et al. carried out a study to evaluate the ocular retention and intraocular delivery of ganciclovir entrapped in niosomes, which were pre-coated with chitosan [43]. The niosomal formulation showed noticeably longer precorneal retention time, which could be contributed to by several factors. For instance, the cholesterol improved the firmness of the niosome lipid bilayer, thereby reducing niosome clearance from the corneal surface [38]. Additionally, the presence of chitosan coating further increased ocular retention of niosomes, due to the interactions of positively charged chitosan with negatively charged components on the corneal surface [43]. Correspondingly, the drug concentrations obtained in the aqueous humour of niosome-treated New Zealand (NZ) albino rabbits were significantly greater than those of other reference formulations. This was associated with a longer time frame for drug release as a result of extended ocular retention, leading to increased drug availability in the aqueous humour [43]. Nanosized niosomes inherently permeate into the ocular membrane better, but also allow even spreading of the niosomal formulation across the application site [43]. Additionally, the presence of a non-ionic surfactant, Span 60, acting as a penetration enhancer, further augmented the drug penetration into ocular tissues [43]. Sustained drug release was also observed for a niosomal formulation (12 h) compared to solution (2 h), which was clearly due to the controlled drug diffusion rate across the niosomal surface. In Akhter’s study, an in vivo ocular irritation test according to Draize methods showed no visual irritation or damaging effect to ocular tissues, suggesting that the niosomal formulation was well tolerated [43].

#### 4.1.2. Solid Lipid Nanoparticles and Nanostructured Lipid Carriers for Ocular Drug Delivery

Solid lipid nanoparticles (SLNs) and nanostructured lipid carriers (NLCs) have been receiving considerable attention as antiviral drug carriers recently. As their name suggests, SLNs are made up of solid lipids at room/body temperature, in combination with surfactants which act as stabilising agents [38,39]. On the other hand, NLCs are synthesised with solid lipid, surfactants, and a small amount of oils (liquid lipids) [38]. Essentially, SLNs and NLCs can be produced at a more reasonable cost and are more stable as compared to liposomes, in addition to having lower acidity and toxicity when compared with certain polymeric nanoparticles [38,44]. In this context, Kumar and Sinha [45] formulated and evaluated SLNs for the purpose of improving ocular delivery of valacyclovir. The SLNs were synthesised using steric acid and tristearin (lipid carrier) plus Poloxamer 188 (surfactant) and sodium taurocholate (co-surfactant). The in vitro release study of the optimised formulation (SLN-6) showed a sustained release profile where approximately >60% of the drug was released over 12 h. This finding substantiated that solid lipid in SLNs assisted in releasing the drug at a controlled rate, by hindering the mobility of the drug in the solid state [38,45]. The percentage of the initial burst release of SLNs (15–27%) was relatively lower than that of solution (>40%). The initial burst release of SLNs was attributed to the release of drug located close to the surface of SLNs [45]. Nevertheless, SLNs were preferred over solution, as the higher initial burst release as observed from solution could result in toxic levels and adverse effects [45]. Apart from that, the ex vivo studies showed enhanced drug permeation and retention of SLN-6 on the excised cornea, when compared with the solution. This was because of their submicron size, and SLNs could cross epithelial barrier of the eye efficiently, thereby increasing the drug penetration and accumulation in the cornea [38,45]. Conversely, the lack of carrier molecule in reference solution constrained the drug penetration into the tightly bound corneal epithelia [45]. Consequently, the in vivo study reported higher valacyclovir ocular bioavailability in the group treated with SLN-6, significantly differing from those treated with solution. The improved ocular bioavailability of SLN-6 was attributed to the better permeation quality of SLNs, as mentioned before. Additionally, the permeation-enhancing effect of sodium taurocholate had contributed to the availability of drug in the aqueous humour [45]. Thus, the valacyclovir distribution from SLNs in the aqueous humour was significantly higher than that in the plasma. The Hen’s Egg Test-Chorioallantoic Membrane (HET-CAM) assay, corneal hydration study, and histopathology observation showed the non-irritancy of the developed SLN-6, most probably due to the use of physiological compatible lipid and excipients [45]. Overall, this study emphasises that SLNs are efficient in targeted drug delivery to ocular tissue, as they could overcome nasolacrimal drainage, which minimises drug availability in the plasma, and in turn, reduces the systemic toxicity of antiviral drugs.

Based on a similar concept, Seyfoddin et al. conducted a study aimed to improve the ocular bioavailability of acyclovir based on SLN and NLC delivery systems [46]. The in vitro release profile showed that drug release from NLCs was faster than for SLNs. A few reasons that contributed to the faster NLC drug release were better wettability and porous nature because of the use of liquid lipid [46]. These features resulted in quicker diffusion and release of the drug from the NLCs. Overall, both NLCs and SLNs showed extended drug release (8 h) compared to the reference solution (4 h). According to the ex vivo corneal permeation study, NLCs exhibited extensive permeation compared to SLNs. This was because the lower zeta potential (negatively charged) of NLCs formed effective interactions with the corneal surface with a positive charge [46]. The presence of liquid lipids may exert a penetration-enhancing effect, enabling increased uptake and transport of NLCs across the corneal barrier [46]. Nonetheless, the corneal hydration level showed all formulations failed to cause any toxicity to the cornea, similar to the result obtained by Kumar and Sinha [45]. In a consecutive work, Seyfoddin et al. conducted a study on the ex vivo and in vivo evaluation of chitosan-coated NLCs for acyclovir ocular delivery [47]. On top of the desirable properties reported in the earlier study, this latest research highlighted that the antiviral efficacy of acyclovir encapsulated in NLCs was superior over that of the conventional acyclovir suspension. Particularly, a 3.5-fold reduction in IC_50_ was observed following encapsulation of acyclovir in NLCs. It was postulated that the higher antiviral efficacy was caused by two mechanisms, including sustained drug release, which would increase the exposure time of infected cells to the acyclovir, leading to a more efficient treatment, as well as virus-induced membrane perturbation which caused the infected cells to become a natural target for NLCs, where NLCs would be efficiently internalised to eradicate the virus within cells [47]. Acyclovir uptake by primary human corneal epithelial cells treated with chitosan-coated NLCs was higher than other non-chitosan-coated agents and suspension. Evidently, the uptake results and antiviral efficacy study were in coherence with each other. More significantly, the MTT cytotoxic study showed that the NLC formulation did not impose any toxic effects on the cultured Vero cells. In summary, the two studies by Seyfoddin et al. are supportive of the promising potential of antiviral SLN and NLC systems.

#### 4.1.3. Advancements of Nanoemulsions for Ocular Drug Delivery

Nanoemulsions have been reported to be successfully formulated as various topical therapeutic agents. Nanoemulsions are described as colloidal dispersion systems composed of two immiscible liquids mixed with emulsifying agents to form a single phase [48,49]. Nanoemulsions have been extensively investigated as ophthalmic drug delivery systems, as they are able to dissolve large amounts of hydrophobic molecules, and prevent the drugs from undergoing hydrolysis and enzymatic degradation [50]. Furthermore, nanoemulsions are favourable in providing higher drug penetration into deeper ocular tissues than conventional emulsions [38]. In this context, Patel and colleagues developed and characterised an acyclovir nanoemulsion in the form of a topical gel, with the purpose of increasing acyclovir solubility and permeability across a biological barrier [50]. The materials used to formulate the nanoemulsion included castor oil, Span 40, and PEG 400, where acyclovir appeared to have better solubility in these ingredients. The in vitro diffusion study performed using a dialysis membrane revealed that the optimised formulation showed approximately 88% drug release within 8 h, conforming to zero-order release kinetics. This phenomenon could be associated with the colloidal dispersion for providing sustained drug permeation across a membrane [13,50]. However, there was a lack research on in vivo ocular bioavailability and efficacy. Hence, more understanding and validation of the nanoemulsion gel for safe and effective delivery via a topical ocular route are required. Another study which employed a nanoemulsion in combination with a thermo-responsive in situ gelling system for acyclovir ocular delivery is discussed in Section 4.5.1. Meanwhile, different approaches of lipid-based nanocarriers in the improvement of delivery strategies in HSK are represented in Table 1.

### 4.2. Polymeric-Based Nanocarriers for Ocular Drug Delivery

Apart from lipid-based ocular delivery, polymeric-based ocular delivery systems also showed favourable outcomes in formulating antiviral drugs targeting HSK. A few examples of different polymer-based delivery approaches such as nanomicelles, nanoparticles and nanosuspension found to be beneficial in combating HSK. The following section will briefly cover the results of different studies performed by incorporating polymers in the nanocarriers intended to be delivered to ocular infections.

#### 4.2.1. Nanomicelles for Ocular Drug Delivery

Nanomicelles are colloidal drug delivery systems made up of amphiphilic surfactant units [31,51,52]. These amphiphilic units can self-assemble and form a nanosized, spherical structure creating a hydrophilic shell and lipophilic core upon reaching the critical micelle concentration (CMC). The CMC is a point where micelle formation starts in the declining surface tension of the system facilitated by surfactants or polymers. Hence, the solubility of hydrophobic drugs is improved in aqueous solution by encapsulating them in the lipophilic core. Nanomicelles are further classified as surfactant nanomicelles and polymeric nanomicelles. In surfactant nanomicelles, the hydrophilic head can be either ionic, zwitterion, or non-ionic, and non-ionic surfactants are believed to be the least toxic among the others [53]. On the other side, polymeric nanomicelles are constructed from block copolymers such as poly (lactic-co-glycolic acid), polyethylene glycol, polylactic acid, polyethylene oxide, and many more. These polymers can conjugate to construct diblock (A-B type), triblock (A-B-A type), pentablock (A-B-C-B-A type), or complex branched type copolymers. Micelle formulation has been actively involved in ocular drug delivery strategies with its benefits of less toxicity, no blurred vision as it exists in an aqueous form, ease of preparation, and better permeability through the corneal epithelial layer, thereby promoting the bioavailability of lipid drugs. Additionally, with the nanosized molecule, the permeability and absorption of the drug are further enhanced. Incorporated polymers in the formulation also permit a sustained release of the drugs. They can maintain the integrity of the micellar structure even in a diluted environment due to lower CMC values.

Recently, a study was conducted by Varela-Garcia et al. comparing encapsulated acyclovir in Soluplus and Solutol polymeric micelles in terms of solubility and permeability in both the cornea and sclera of the eyes [54]. Acyclovir has a low water solubility characteristic and was marketed in the form of an ocular ointment. In this study, the recorded solubility of acyclovir was 1.02 mg/mL, depending on the pH environment [55]. Soluplus and Solutol were selected to improve the solubility of acyclovir. Soluplus is an authorised biodegradable block copolymer with low toxicity, biocompatibility, and a low CMC value of approximately 7.6 mg/L, allowing high stability of micelles [56]. Soluplus also has in situ gelling properties, which would enable retention of the formulation on the cornea and permit permeability [54,56]. Both Soluplus and Solutol have a similar hydrophilic–lipophilic balance of 16 and 16–18, respectively. However, Solutol was discarded from the following study because Soluplus showed a better acyclovir solubility than Solutol in both water and phosphate-buffered saline (PBS) medium. Evidence revealed that acyclovir solubility increased 2-fold in both water medium and in PBS. Moreover, the micelle–water partition coefficient for Solutol dispersion was below 1, indicating that acyclovir was more soluble in water than the core space of the polymeric micelles. Therefore, the permeability study was proceeded by comparing acyclovir in Soluplus micelles (ACV-SMs) and the aqueous solution of acyclovir (ACV-Aq). A bovine cornea and sclera permeability assay was performed in Franz diffusion cells. The results have shown that the permeation in the sclera was much higher compared to the corneal. The study explained the permeability with a few parameters. Firstly, the acyclovir amount that passed through the cornea and sclera layer and accumulated in the receptor chamber was recorded. In the cornea, ACV-SMs levels were 5.3-fold higher than ACV-Aq and 3.3-fold higher in the sclera. This result indicated that the Soluplus micelle can carry the drug and permeate through the cornea and sclera by overcoming the corneal tight barriers. Next, the accumulated amount of acyclovir in the cornea and sclera was recorded. In the cornea, ACV-SMs levels were 5.7-fold higher than ACV-Aq and 6.8-fold higher in the sclera. The improvement due to the permeated micelles can be retained in both the cornea and sclera layers after permeation. Moreover, the permeability flux also showed remarkable improvement in ACV-SMs with levels 2.8-fold higher than ACV-Aq in the cornea and 3.4-fold higher in the sclera. The magnificent permeability of the sclera was explained by its porous structure made up of collagen and polysaccharide fibres, where the diffusion of macromolecules and nanosized molecules happened easily [54]. Besides, the ACV-SMs displayed accumulation in the sclera, allowing most drug molecules to slip through it readily and facilitate entry to the posterior part of the eye. An additional permeability test comparing Soluplus 20%, Soluplus 12%, and an aqueous form also favoured the Soluplus polymer formulation. As the study failed to compare Soluplus and Solutol polymers, another study carried out by Sun et al. comparing the polymers as proposed in the Varela-Garcia et al. study concluded that the Soluplus micelles showed better corneal permeation than Solutol micelles [56]. The micellar structure of Soluplus was clarified to have an active endocytosis uptake mechanism when entrapping the micelles with a fluorescent dye (Cou-6) [56,57].

Another study performed by Vadlapudi et al. also found excellent permeability. Vadlapudi et al. formulated surfactant nanomicelles with biotin-12-hydroxystearic acid-acyclovir (B-12HS-ACV) and two non-ionic surfactants, d-α-tocopheryl polyethylene glycol 1000 succinate (vitamin E TPGS) and octoxynol-40 prepared by a solvent evaporation/film hydration method [58]. Vitamin E TPGS was FDA approved and has been a popular surfactant in formulating prodrug carriers, micelles, liposomes, and many more. It also functions as an efflux transporter inhibitor to promote drug absorption and bioavailability of the lipid prodrug B-12HS-ACV [59]. On the other hand, octoxynol-40 was added to the formulation to improve the nanomicelle structure and lower the CMC value of vitamin E TPGS, thus creating a more stable nanomicelle integrity [60]. The released pattern of the developed formulation was much more stable compared to the prodrug dissolved in ethanol solution (control). The nanomicelle formulation released the prodrug for 4 days, while the ethanol solution released the prodrug within 6 h [58]. The developed nanomicelles appeared to be as clear as water. Hence, it was better than a suspension formulation as it did not disrupt vision. Moreover, the nanosized micelle characteristic allowed permeation via transcellular and paracellular pathways [58]. Notably, the nanomicelle formulation did not show a significant burst release of the prodrug thanks to the concrete integrity of the mixed micelle formulation [58]. Burst release is a major disadvantage in formulating micelles, and it can be overcome by improving the encapsulation efficiency. The encapsulation efficiency, in this case, was approximately 90%.

All the formulated nanomicelles in both studies were proven to be biocompatible. The compatibility testing was performed by a chorioallantoic membrane (CAM) in the Soluplus polymeric micelle study, whereas human corneal epithelial cells (HCECs) were used in the surfactant nanomicelle study. CAMs of fertilised hen eggs resembled the conjunctiva of rabbit models and was proposed as an alternative for the Draize test [54]. The developed Soluplus polymeric micelles showed an absence of haemorrhage, vascular lysis, or the coagulation of CAM vessels, suggesting no irritation upon application. Besides, HCECs were chosen to be the in vitro cell culture model due to their ability to express cytokines and chemokines in response to inflammation. The cytotoxicity measurements were determined by the amount of formazan formed. During the incubation of HCECs with blank and prodrug-encapsulated nanomicelles, the formazan formation was not significantly affected. On top of that, both blank and nanomicelle formulations did not display significant alterations of cytokine levels [58]. In conclusion, both formulations were justified to be non-toxic in the eyes and disclosed the possibility of a nanomicelle strategy in improving acyclovir in treating HSK conditions.

#### 4.2.2. Polymeric Nanoparticles and Nanosuspensions for Ocular Drug Delivery

Polymeric nanoparticles are colloidal drug delivery systems with a size range of 10–1000 nm, categorised as nanocapsules and nanospheres depending on the preparation method and the composition of the polymers [61]. Common polymers used in ophthalmic preparations include natural polymers, semisynthetic polymers, and synthetic polymers [62,63]. They provide several advantages, such as (1) less ocular irritation; (2) improved corneal retention time, thus better drug absorption; (3) controlled drug release and hence the avoidance of frequent administration; and (4) drug protection against degradation. Implementation in a suspension system is required to topically administer nanoparticle formulations to the eyes. The stability of the drug dispersion is facilitated by appropriate stabilising agents, such as surfactants or polymers. Recent research on acquiring polymeric nanosuspensions targeting HSK appeared to be utilising polymers such as chitosan and poly (lactic-co-glycolic acid) (PLGA). The details of the study are explained in the subsequent paragraph.

Similar studies carried out by Rajendran et al. and Selvaraj et al. on characterising the chitosan nanoparticle formulation incorporating acyclovir revealed biphasic release characteristics with a short rapid burst release of acyclovir and continuous release over time [64,65]. The initial burst was described by the drug release near the nanosphere surface, followed by continuous drug release within the nanosphere. The formulation possessed a zero-order release rate and showed a non-Fickian diffusion mechanism. It is essential to highlight that the interaction between the amino group of chitosan with acyclovir was observed during the study, which prolonged the release of the drug from the formulation. Besides, Selvaraj et al. also compared the chitosan nanoparticles with marketed acyclovir ointment. The results showed a remarkable improvement in accumulated drug release over time in the chitosan nanoparticle formulation of up to 24 h [65].

**Figure 3:**
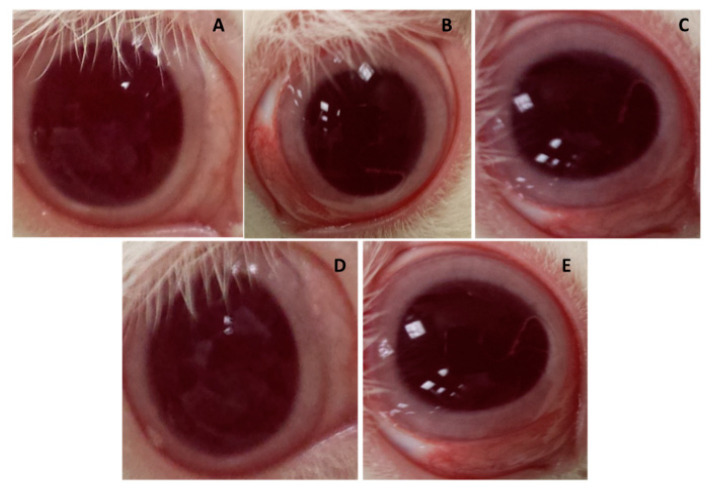
Ocular irritation study conducted by Alkholief et al., revealing (**A**) normal condition of rabbit eye; (**B**) mild redness and inflammation in the conjunctival area by the first hour post-administration; (**C**) increased redness and inflammation by the third hour; (**D**) reduced redness and inflammation by the sixth hour; (**E**) complete reduction of redness and inflammation by the twelfth hour, adapted with permission from [66], Elsevier, 2019.

On the contrary, another acyclovir treatment formulated in PLGA nanoparticles employed vitamin E TPGS as a stabilising agent also displayed a biphasic release mechanism with rapid release followed by 72 h of controlled release [66]. In this study, the release rate followed the first-order release and obeyed Fick’s diffusion mechanism law instead. Alkholief et al. conducted a transcorneal permeation study to evaluate the mucus-penetrating capacity in fresh albino rabbits’ corneas by exposing them to both the acyclovir solution and ACV-PLGA-TPGS nanoparticles. The permeability coefficient was $\left(18.627\pm 0.0077\right)\times 10^{-2}\mathrm{cm}/h$ in ACV-PLGA-TPGS nanoparticles while for the acyclovir solution it was $\left(13.063\pm 0.0049\right)\times 10^{-2}\mathrm{cm}/h$. The author explained that vitamin E TPGS contributed its inhibitory effect on the P-glycoprotein efflux transporter. On top of that, as mentioned in the Vadlapudi et al. study, its self-assembly property improved acyclovir solubility via micellar formation to ultimately ameliorate the penetration process via inter- and intracellular pathways [59,66,67]. A hypothesis was made that pH affects the permeation of acyclovir, as both the pH level of the acyclovir solution and ACV-PLGA-TPGS nanoparticles shifted towards the neutral pH of the tear fluid. The pKa values of acyclovir of 2.52 and 9.35 implied that it was a weak acid and base itself. The pH shifted to 7.4 upon contact with the eye, where acyclovir became an unionised form, thereby showing improved permeability [68]. Furthermore, with the enhanced permeation of acyclovir in the nanoparticle formulation, validation of acyclovir retained in the aqueous humour of the albino rabbit eyes was further explored by ultra-high-performance liquid chromatography-ultraviolet (UPLC-UV). The results showed that the acyclovir concentration in the solution formulation was available for up to 6 h, while in the PLGA-TPGS nanoparticle formulation, it was available for up to 24 h. The pharmacokinetic parameters have shown that the $AUC_{0-inf}$ of the ACV-PLGA-TPGS nanoparticle formulation was 2.8 times higher than that of the acyclovir solution and the $mean\;residence\;time_{0-inf}$ of the ACV-PLGA-TPGS nanoparticle formulation was 2.2 times longer than that of the acyclovir solution. The prolonged residence time was mainly due to the presence of vitamin E TPGS with a positive zeta potential, creating electrostatic forces with negatively charge glycoprotein found in the mucin layer. An ocular irritation test was also conducted by Alkholief et al., where the sample was administered three times a day for 10 days (Figure 3). In the first hour after administration, mild redness was observed, and this increased further by the third hour. The redness then subsided by the sixth hour and completely disappeared by the twelfth hour. During the observation, the authors pointed out that one animal experienced mucoid discharge (grade 1). No tissue damage to the conjunctiva was observed, suggesting that the ACV-PLGA-TPGS nanoparticles were relatively safe, non-toxic, and not irritating to the eyes. Table 2 is the summarised outcomes of all the polymeric-nanocarrier approaches in managing herpes keratitis.

### 4.3. Prodrug Approach for Ocular Drug Delivery

Prodrug technology has turned out to be a justified tool for ocular drug development, as it has considerable potential to improve the fate of drugs in ocular tissues. A prodrug is an inactive form of a therapeutic agent that is converted in the ocular tissues into an active drug or parent drug by enzymatic action [31]. The aim is to optimise the physicochemical properties of drugs to improve their solubility, in vivo stability, and pharmacokinetic features to impede premature metabolism. This can achieve targeted drug delivery and reduce the incidence of unwanted effects, and hence boost patient compliance [69,70]. It has been recommended that some ideal characteristics must be fulfilled while developing prodrugs, such as being non-toxic, chemically stable, and remaining inactive before reaching the site of action [69,70]. The functional groups used in ophthalmic prodrug designs are carboxylic, carbonyl, amine, amide, hydroxyl, esters, phosphates, oximes, and carbamates [71]. Moreover, several transporters have been found in the ocular tissues, which include influx transporters, efflux transporters, peptide transporters, amino acid transporters, vitamin transporters, glucose transporters, organic anion transporters, and organic cation transporters [72]. The influx transporters play an important role in delivering essential nutrients and therapeutic agents across the ocular barriers [71]. Hence, a prodrug can be designed with specificity for the transporter, known as a transporter-targeted prodrug. In this context, Katragadda et al. studied the ocular pharmacokinetics of amino acid prodrugs of acyclovir (ACV) including γ-Glutamate-ACV (EACV), L-Serine-ACV (SACV), L-Valine-ACV (VACV), L-Alanine-ACV (AACV), and L-Isoleucine-ACV (IACV) in rabbits using a topical well infusion and microdialysis method. SACV and VACV showed two-fold higher levels in the area under concentration time curve (AUC) and maximum aqueous humor concentration (C_max_) of the prodrug and regenerated ACV (total concentration) compared to ACV. Yet, the total concentration of the last time point (Clast) of VACV was lower than that of SACV due to the longer half-life of SACV and lower enzymatic stability of VACV, which leads to rapid regeneration and elimination of ACV, although it has comparable AUC to SACV [73]. A similar result was obtained by another group of researchers, where SACV exhibited better permeability and a superior HSV-1 inhibition effect in rabbit cornea epithelial cell culture relative to ACV [74]. In short, the ocular bioavailability of acyclovir can be improved by using an amino acid prodrug approach. 

Apart from that, dipeptide monoesters were found to have a greater affinity to peptide transporters present on the corneal epithelium [75]. In a study, Majumdar and co-authors evaluated dipeptide monoester ganciclovir (GCV) prodrugs, such as tyrosine-valine-GCV (YVGCV), glycine-valine-GCV (GVGCV), divaline-GCV (VVGCV), and valine-GCV (VGCV) topical ophthalmic solution for ocular bioavailability. According to the results, all the prodrugs exhibited higher aqueous solubility compared to GCV. Particularly, VGCV and VVGCV showed seven- to eight-fold higher transcorneal permeability than GCV due to greater lipophilicity and corneal peptide transporter (PepT1)-mediated translocation across the corneal epithelium. An in vivo efficacy study showed that 1% VVGCV has better therapeutic activity against HSV-1 epithelial keratitis compared to 1% trifluridine in the rabbit ocular model. Significantly, the rebound elevation in slit-lamp examination (SLE) score was observed in the trifluridine treatment group but not in the VVGCV treatment group. VVGCV demonstrated better corneal permeability, chemical stability, high aqueous solubility, and in vivo anti-HSV-1 activity [75]. Similarly, another in vivo comparative study reported that YYGCV and VVGCV exhibited enhanced permeability bioavailability compared to VGCV and GCV. However, the area under the concentration–time profile (AUC_infinity_) of regenerated GCV from YVGCV was found to be the largest among the other prodrugs in this study. According to the data, the AUC_infinity_ of the regenerated GCV from YVGCV is 8.6-fold larger than for GCV, whereas for VVGCV, it is 1.8-fold higher than GCV, suggesting that YVGCV has a better pharmacologic profile compared to VVGCV [76]. Markedly, the dipeptide monoester prodrug of GCV has an affinity towards PepT1 with better permeability and therapeutic efficacy.

On the other hand, a sodium-dependent multivitamin transporter (SMVT)-targeted prodrug combined with a lipid prodrug approach was developed by Vadlapudi et al. to boost the cellular absorption of ACV [77]. The researchers compared the cellular uptake of an ACV prodrug, such as lipid prodrugs (R-ACV and 12HS-ACV), biotin-ricinoleicacid-acyclovir (B-R-ACV), biotin-12hydroxystearicacid-acyclovir (B-12HS-ACV), and biotinylated but non-lipidated prodrug (B-ACV). The uptake study was performed using human corneal epithelial (HCE) cells and showed that B-R-ACV and B-12HS-ACV have higher intracellular drug accumulation than B-ACV, R-ACV, and 12HS-ACV. The enhanced uptake could be due to carrier-mediated transport by the SMVT and increased lipophilicity. Moreover, in vitro antiviral testing showed that B-R-ACV and B-12HS-ACV have higher potency against both HSV-1 and HSV-2. [77]. As a result, the combination of a transporter-targeted prodrug approach and lipid prodrug approach can further enhance the drug uptake as well as the antiviral activity.

A few of the prodrug approaches against HSK are represented in Table 3. To conclude, the use of prodrugs has the potential to enhance ocular bioavailability by modifying the physicochemical properties of the drugs, which could be a promising approach in the treatment of HSK. 

### 4.4. Peptide Delivery Approaches for Ocular Drug Delivery

Proteins and peptides are naturally occurring biological molecules and provide benefits in drug delivery systems, such as high potency and specificity and low toxicity [78]. However, these molecules possess some drawbacks owing to their high molecular weight, poor permeation, poor stability, and low circulation half-life, resulting in poor bioavailability and are insufficient to achieve optimal treatment outcomes [78,79]. Owing to that, peptides are ideally formulated as topical formulations for treating keratitis. Extensive research has been carried out for the development of peptide delivery, and various antimicrobial peptides (AMPs), also known as host defense peptides, have been found to have potential in treating ocular infections. AMPs are the fundamental components of the innate immune system and are generally positively charged and found in a wide variety of organisms, from human to microorganisms [80,81]. The antimicrobial effect seems to rely on the electrostatic interaction between the negatively charged cell membranes and positively charged AMPs, which leads to the disruption of the cell membrane [80]. Cathelicidin (LL-37) is one of the AMPs which has been shown to have the ability to hinder the growth of HSV. In vitro studies performed by Gordon et al. showed that a concentration of 500 µg/mL of LL-37 possesses anti-HSV-1 activity, where it produced a reduction significantly more than 100-fold in HSV-1 viral load in a direct inactivation time–kill assay compared to PBS (control) and scrambled peptide [82]. Similarly, a more recent study was conducted to compare the sustained release of LL-37 from nanoparticle–hydrogel corneal implants with human corneal epithelial cell (HCEC)-produced LL-37 and demonstrated that it could reduce the viral binding. However, LL-37 was not able to completely clear the virus from already infected cells, although it showed anti-HSV-1 activity [83]. This can be explained by the mechanism of LL-37 where it inhibits HSV-1 infection by entry inhibition and prevents viral cell attachment [84].

**Figure 4:**
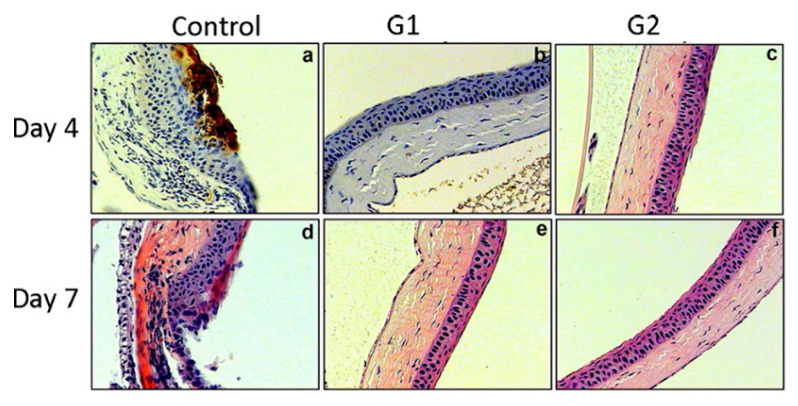
A mouse model of corneal keratitis was given 100 μL of 0.5mM of G1 (peptides with alternating charges), G2 (peptides with repetitive charges), and designated peptide (control) as a prophylactic eye drop followed by the inoculation of HSV-1. Immunohistochemistry was carried out using anti-HSV-1 glycoprotein D (gD) polyclonal antibody on day 4 and day 7 post-infection to detect the HSV-1 gD expression in the cornea. (**a**) chronic inflammation was observed together with significant brown staining in the pretreated cornea with control on day 4, which was indicating the expression of HSV-1 gD; (**b**) on day 4, HSV-1 gD staining was not detected in the cornea pretreated with G1 peptide; (**c**) on day 4, HSV-1 gD staining was not detected in the cornea pretreated with G2 peptide; (**d**) the staining was disappeared in the cornea pretreated with control on day 7 but the damage of the corneal epithelium was observed; (**e**) on day 7, HSV-1 gD staining was not detected in the cornea pretreated with G1 peptide and the corneal epithelium remained intact; (**f**) on day 7, HSV-1 gD staining was not detected in the cornea pretreated with G2 peptide and the corneal epithelium remained intact. The results indicated G1 and G2 significantly blocked the entry of HSV-1, adapted with permission from [85], American society for biochemistry and molecular biology, 2011.

Heparan sulfate (HS) is abundantly found on cell surfaces and 3-O-sulphated HS can be produced through an enzymatic modification of HS [85]. 3-O-sulphated HS is a receptor that is essential for the entry of HSV-1 glycoprotein D (gD), where their interaction promotes fusion pore formation during viral entry [15,85]. AMPs can be used to inhibit this interaction to prevent viral entry. Notably, their antiviral effect is associated with the ability to suppress the cell-to-cell spread of viruses across tight junctions or impede the formation of giant cells [86]. Tiwari and co-workers identified G1 (peptide with alternating charges) and G2 (peptide with repetitive charges) that bind specifically to heparan sulphate (HS) and/or 3-OS HS and block HSV-1 entry by screening the M13-phage display peptide library. The outcome revealed that the G1 and G2 peptides inhibited HSV-1 entry with half maximal inhibitory concentration (IC_50_) value of 0.02 to 0.03 mM. They also showed that the severity of keratitis was significantly reduced in mouse corneas when 100 μL of 0.5 mM G1 and G2 peptides were administered prophylactically (Figure 4). Additionally, the study showed the in vivo significance of HS and 3-OS HS as a coreceptor of HSV-1 [85]. 

**Figure 5:**
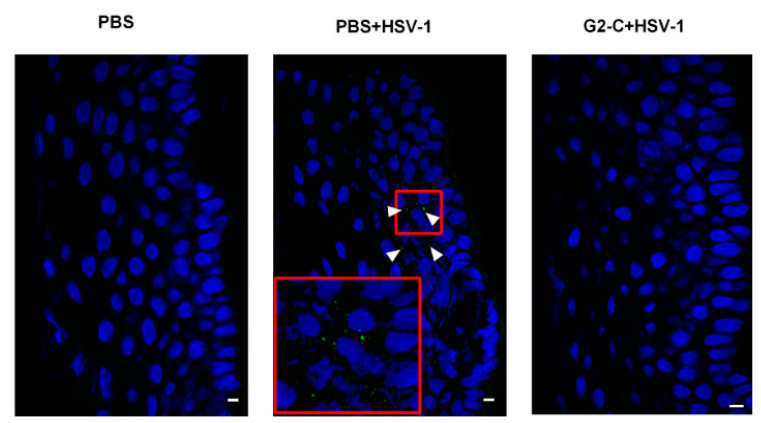
The sections from pig corneas received indicated treatments, where the epithelial cells are shown in blue, while the virus is shown in green. The phosphate-buffered saline (PBS) + HSV-1-treated cells showed the presence of virus while G2-C + HSV-1-treated cells did not. It could be deduced that the peptide, G2-C, released from the contact lens was effective in inhibiting HSV-1 infection, adapted from [87], ARVO Journals, 2016.

A more recent study improved the stability of G2 peptide by attaching a cysteine residue at its C-terminus (G2-C) and loading into a contact lens. The modified G2 peptide has a similar effect in decreasing HSV-1 entry as G2. Study results revealed that G2-C was released through contact lenses in an extended-release manner. The pretreated human corneal epithelial (HCE) cells with G2 peptide showed less HSV-1 entry compared to the pretreated HCE cells with PBS (control). The G2-C lens was also found to be effective in inhibiting HSV-1 entry in both human and pig corneas ex vivo (Figure 5) and mouse in vivo models [87]. Indeed, G2 peptides have the ability to suppress ocular herpes and the release can be prolonged with the use of contact lenses.

The human apolipoprotein E (apoE) gene (APOE) has three allelic types, including ε2, ε3, and ε4 [88]. The apoE ε4 type has been recognised as a risk factor in HSV infection [84,88]. Accordingly, therapeutic agents can be developed to mimic apoE action against the infection. The dimer peptide derived from human apolipoprotein (apoEdp) demonstrated antiviral activity against HSV [88,89]. Bhattacharjee et al. conducted in vivo evaluations of therapeutic efficacy with 1% apoEdp using a mouse ocular model of herpetic stromal keratitis. The 1% apoEdp was found to be as effective as 1% trifluridine in reducing the incidence and severity of HSK. In addition, the expression of proinflammatory cytokines IL-1 α, IL-1 β, IL-6, TNF α, IFN-γ, and vascular endothelial growth factor (VEGF) was found to be downregulated compared to the control [88]. Thus, this study has suggested that apoEdp possesses both anti-HSV-1 and anti-inflammatory effects. Subsequently, another study was carried out to investigate the effect of apoEdp against thymidine kinase (TK)-positive and TK-negative HSV-1 in rabbit ocular models. As TK-negative virus is resistant to nucleoside analogue antivirals like trifluridine, therefore, 1% apoEdp was compared with 1% trifluridine in a TK-positive HSV-1 group and 1% apoEdp was compared with 3% foscarnet in a TK-negative HSV-1 group. The results obtained pointed out that apoEdp was as effective as trifluridine and foscarnet in reducing the severity of keratitis, suggesting that apoEdp has the potential to be used in the treatment of HSK with resistant strains [89]. In another study, authors emphasised the stability concern related to peptide delivery and stable formulation development for peptides. Antimicrobial peptides became popular for infectious ocular keratitis treatment to avoid bacterial resistance caused by classical drug treatment. In this regard, Terreni et al. developed freeze-dried solid matrices containing hLF 1-11, a synthetic antimicrobial peptide for the treatment of ocular keratitis. The formulation showed better stability of the peptide and effective antimicrobial activity with sustained release of the peptide [90].

Despite the fact that peptide delivery has a favourable outcome in the treatment of herpetic keratitis, it is challenging to formulate and deliver peptide therapeutics due to their large size, poor permeation and poor stability [78]. Moreover, this approach is time-consuming, and it is costly to identify an ideal peptide [80]. Different peptide deliveries in the treatment of HSK are summarised in Table 4. 

### 4.5. In Situ and other Approaches for Ocular Drug Delivery

The in situ gelling system is amongst the most well-known novel medicament delivery systems. These formulations exist in solution form before administration into the body and undergo phase transition into the gel form. They are stimulated by various stimuli such as the alteration of temperature, pH, solvent exchange, ultra-violet irradiation, and the existence of specific ions. Other than that, in situ gels are classified according to the route of administration. In situ gels are mainly delivered into the patient’s body through the oral, ocular, rectal, vaginal, nasal, and parental route [91]. Subsequent sections of this article will emphasise in situ gel-based ocular deliveries projected for the treatment of eye infections.

#### 4.5.1. In Situ Ocular Gel for Ocular Drug Delivery

The in situ ocular gel is one of the advanced drug delivery systems to overcome the difficulty of delivering the therapeutic agents to the affected site in the eye. It consists of environmentally sensitive polymers that undergo structural changes under the influence of an external stimulus. These polymers are in solution form before administration into the eye. Once it is instilled into the eye, it undergoes rapid gelation as the environmental conditions, specifically temperature, pH, and ionic strength, are altered [92].

One of the bonuses of incorporating herpes keratitis drugs into an in situ gel ocular formulation is to lengthen the residence time of the active drug on the cornea. According to Varela-Garcia et al., the complex viscosity value measured for ocular in situ micelles at 35 °C in water was 0.50 Pa·s, whereas in phosphate-buffered saline (PBS), it was 3.12 Pa·s, suggesting that the viscosity of ocular in situ micelles increases when in contact with saline at a temperature of 35 °C. As the viscosity of the solution increases after the change of temperature, pH, and ions in another environment, the residence time of the drug on the surface of the cornea also lengthens [54]. 

Additionally, the advantage of using an ophthalmic in situ gel formulation in the management of herpes keratitis includes controlling the release of the active ingredients onto the cornea. An in vitro drug release study of an ophthalmic nanoparticle formulation and ophthalmic in situ nanoparticle formulation had been carried out by Yang et al. using the dialysis bag method. In the in vitro release study, the release rate was observed to be 5.60, 4.47, and 5.18 $h^{-1/2}$, respectively, for L-Val-L-Val-ganciclovir (LLGCV), L-Val-D-Val-ganciclovir (LDGCV), and D-Val-L-Val-ganciclovir (DLGCV) nanoparticle formulations. The ophthalmic nanoparticle formulation was in the first-order release and followed the Higuchi model and Fickian diffusion mechanism. In short, the release pattern of the active ingredient depends on the concentration and on an unerodable matrix through diffusion that obeys Fickian laws. Therefore, a biphasic release is observed in nanoparticle formulations. On the other hand, release rate constants from nanoparticles incorporated in in situ ophthalmic gels were measured and recorded as 0.538, 0.559, and 0.570μg/h for LLGCV, LDGCV, and DLGCV, respectively. Moreover, the formulation of nanoparticles in thermo-responsive gels shows a zero-order drug release behaviour within a period of 28 days [93]. Thus, ophthalmic nanoparticle in situ gels ensure a constant release of the active drug to the site of action, minimise concentration fluctuation, and reduce the rates of local ocular toxicity in herpes keratitis patients.

Similarly, an in vitro study was conducted by Mahboobian et al. with a comparison between an ocular acyclovir solution and ocular acyclovir in situ nanoemulsion formulation using a dialysis bag method. The results showed that the in vitro release efficiency of all ocular acyclovir in situ nanoemulsion formulations measured after 6 h was between 74.44 and 80.78%, whereas it was 100.54% for the ocular acyclovir solution. Samples were taken after 30 min, and nearly all of the acyclovir in the solution was released, whereas acyclovir in situ nanoemulsion formulations released only about 55–60% of the total amount of acyclovir. This indicated that in situ nanoemulsion formulations had successfully delayed the release of acyclovir [94]. Likewise, Li et al. made a comparison between the rate of release of acyclovir in conventional eye drops and an ion-activated in situ ophthalmic gel using the dialysis bag method. The outcome showed that nearly 95% of acyclovir was released after 0.5 h, and then all acyclovir particles were released soon after. On the contrary, about 17% of acyclovir was released from the ophthalmic in situ gel formulation after 0.5 h, and approximately 80% of the total loaded acyclovir was released after 6 h. Thus, all evidence concluded that the drug took a longer time to be released into the site of action using the ocular in situ gel formulation [95]. Hence, the ocular drug release was retarded using the in situ gel formulation. This helps to guarantee a prolonged pharmacological effect of antiviral drugs, subsequently decreasing the dosing frequency and the amount of drug needed to be administered to the patients.

As more active drug stays on the cornea, the local drug absorption and local bioavailability also increase. As a result, the therapeutic effect of the drug improves in patients with herpes keratitis. Likewise, a research article regarding the treatment of viral eye infection using acyclovir with the application of a dual mechanism in situ gelling system written by Ranyadevi et al. was published. In this study, the comparison of a nanoparticle formulation and nanoparticles incorporated into a combination of Pluronic F-127 with a Carbopol in situ gel system was made using the dialysis membrane method. A percentage of 15 to 40% of the total loaded acyclovir was released within 1 h after administration and, subsequently, an increase in drug release was measured of between 60% and 100% within 8 h from the nanoparticle formulation. The nanoparticle formulation had successfully controlled and delayed the acyclovir release for more than 8 h. The release of drug particles within the first hour may be because some drug particles were not successfully entrapped in the nanoparticle, causing a non-sustained drug release. More drug release occurred later because a larger amount of drug particles was liberated from the nanoparticles. As for nanoparticles incorporated into an in situ gel formulation, 2.75 to 4.22% of acyclovir was released within 1 h after administration and then the maximum level of 6.96 to 32.31% was released after 8 h. The conclusion of these findings was that a greater and more sustained release was seen in the nanoparticles incorporated in an in situ gel formulation than the nanoparticle only formulation. This can be explained by the drug particles that failed to be entrapped in nanoparticles which could still be entrapped in the in situ gel dispersion media to sustain its release [96].

Aside from that, the in situ ophthalmic formulation is proven to cause less or no irritation and itching in patients after instillation into their eyes infected by HSV. A HET-CAM test conducted by Mahboobian et al. revealed that the cumulative score for the acyclovir in situ ocular gel formulation was 0.33. It falls within the range of the non-irritant category, between 0.0 and 0.9. Both results showed that the in situ formulation clearly caused less or no irritation and it is non-toxic for ocular drug delivery use [94]. Correspondingly, Li et al. conducted an ocular irritation assay in healthy NZ albino rabbit eyes with ion-activated in situ ocular gel. The evidence indicated that no damage or abnormal clinical signs were observed in the eyes of the albino rabbits. The results indicated that the formulation did not irritate the rabbit eyes [95]. Similarly, referring to a controlled trial carried out by Lin et al., it was indicated that the numbers of patients reporting slight eye irritation and itching in second, third, and fourth visits were lower in the ganciclovir in situ ocular gel group [97]. Therefore, the patients’ adherence to their treatment plan could be improved if no or less unpleasant experiences, like blurred vision and eye irritation, are encountered after using the in situ gel formulation. Similarly, Kumar and colleagues carried out the most recent study on formulating valacyclovir in the pH-responsive in situ gel system by utilising Carbopol 940 and showed sustained release of the formulation. The in vitro diffusion of valacyclovir performed in Franz diffusion cells revealed that the drug followed zero-order release for up to 8 h. However, the authors did not discuss the biocompatibility of the formulation or the bioavailability of the valacyclovir after application. Valacyclovir is currently available in an oral dosage form for off-label treatment and is not marketed as a topical formulation. Therefore, further evaluation of this active ingredient should be performed to determine whether it provides the same or better efficacy in managing HSK. Moreover, the compatibility of the formulation should also be evaluated to ensure it is harmless when administered to human eyes [98].

Previously, Kapadia et al. also developed acyclovir with niosomes entrapped in a pH-responsive hydrogel. Instead of Carbopol 940, Carbopol 934 was incorporated as the gelling agent [99]. The authors compared Span 20 and Span 60 combined with cholesterol and discovered that a ratio of 7:6 of Span 60: cholesterol possessed acceptable entrapment efficacy. The authors explained that the entrapment efficacy varied with the ratio of surfactant to cholesterol. As the cholesterol reached the maximal concentration, it raised the microviscosity of the membrane, thereby forming a more rigid bilayer with the surfactant. Consequently, it disrupted the regular bilayers and lost its drug entrapment ability. Span 60 was selected over Span 20 due to its longer alkyl chain length, ultimately contributing to better drug entrapment. Interestingly, the authors compared the release kinetics of (D1) acyclovir in niosomes only entrapped in hydrogel and (D2) acyclovir in both niosome and hydrogel systems and revealed that the released was much slower in D1 compared to D2. In D1, the drug available only in the core of the niosomes passed through two barriers: the vesicle layers first, then through the hydrogel matrix, showing only 52% cumulative drug release after 16 h. On the other hand, D2 had acyclovir available in both the niosome and hydrogel system and allowed most of the available drug to be released from the hydrogel matrix first, whilst the drug in the niosomes acted as a reservoir to be delivered in a sustained manner, showing 76.5% cumulative drug release after 16 h. In other words, D2 showed 1.47-fold greater cumulative drug release compared to D1, while providing a reservoir for prolonging drug release. The initial release of D2 appeared to be rapid compared to D1, as the system was exposed to the external environment, unlike D1, which was required to cross two barriers. In summary, the combination system proved to have better sustained release, thereby reducing frequent drug administration and improving patient compliance. Besides, they also carried out biocompatibility testing under a Draize protocol and reported that the combination system was safe, with no irritancy recorded for both D1 and D2. Nevertheless, a permeability investigation was suggested to further explore the overall drug permeability of this combination system and the bioavailability of the acyclovir remaining in the ocular chamber to understand the whole picture of the improvement of this approach. In brief, the in situ gelling systems are viable approaches for the enhanced ocular delivery of antiviral medications. A summary of various ocular smart delivery systems based on in situ gel are depicted in Table 5.

#### 4.5.2. In Situ Minitablets for Ocular Drug Delivery

An ocular minitablet is another useful solid dosage drug carrier for the ocular route of administration. It has a diameter of 2 to 4 mm and is inserted into the conjunctival sac of the eye [100]. Ocular minitablets also exist in in situ formulations. Once the in situ minitablet contacts the lacrimal fluid on the eye, it undergoes rapid gelation or slow dissolution, releasing the active ingredient at the targeted site. The advantages of ocular in situ minitablets include that it is biodegradable, it is eliminated naturally, and manual removal is not needed. After the minitablets undergo phase transition into a gel phase, the residence time for the formulation on the infection site increases [101]. As a result, the dosing frequency is decreased and this improves the compliance, especially of elderly and forgetful patients and patients with a busy working schedule [100]. Moreover, the local bioavailability of the therapeutic agents improves as it is slowly removed by body defense mechanisms [101].

Refai et al. performed an in vitro release study of ocular in situ acyclovir minitablets of many different polymers. Each tablet was placed in a bottle containing phosphate-buffered saline (PBS) of the pH adjusted to7.4 and the temperature was adjusted to around 35 °C. The findings showed that chitosan had the lowest release rate among other polymers, like sodium carboxymethyl cellulose (NaCMC), hydroxypropyl methylcellulose (HPMC), xanthan gum, and carbomer, with the same concentration. This is because the swelling of the pH-sensitive chitosan minitablet occurs after both the negative charges of the polyacid and positive charges of the ammonium groups neutralise each other and become free of negative and positive charges in a solution. However, neither a lack of negative charges nor positive charges can be achieved easily in normal circumstances. Therefore, the swelling rate declines and delays the formation of the gel layer, which plays an essential role in regulating the drug release. Apart from that, an in vivo study was conducted on NZ rabbits. One chitosan minitablet and the marketed Zovirax^®^ eye ointment were administered into each lower conjunctival sac in all rabbits. Care was taken not to irritate the eye or touch the corneal surface of the eye. The rabbits were sacrificed, and their corneas were separated at the time intervals of 1.5, 3, 5, and 7 h. Chitosan polymer was chosen because it shows good sustained release properties and acceptable bio-adhesion properties as well as minimising irritation due to dryness. The results showed that the chitosan minitablet obtained a superior acyclovir peak concentration in the cornea in comparison to the marketed Zovirax^®^ eye ointment. Likewise, chitosan minitablets remarkably improved the local absorption of acyclovir on the cornea in comparison to the marketed Zovirax^®^ eye ointment, where the area under the curve (AUC) values were 713.74 µg/g.h for the acyclovir minitablet and 563.88 µg/g.h for the marketed acyclovir ointment. The permeation and bioavailability of the acyclovir chitosan minitablet were higher due to the mucoadhesive effect of the chitosan when interacting with the mucous layer on the corneal surface. Besides, an in vivo study on Bokhara Trumpeter pigeons with bilateral ocular herpes infection was performed on two groups. A chitosan minitablet was administered to the first group, whereas no treatment was given to another group. The pigeons of the first group were observed to manifest less lacrimation and redness of the eyes 24h after administration of the chitosan minitablets. Within 48h of administration of the chitosan minitablets, complete recovery of symptoms was seen and the pigeons showed no signs or symptoms of recurring infection for the next three months. In contrast, the untreated group exhibited no improvement in their manifestation [101]. The clinical efficacy of the acyclovir in the pigeon group using ophthalmic in situ minitablets was better than the group without treatment because of the local bioavailability in the group that received the in situ minitablets as treatment.

#### 4.5.3. Ocular Inserts for Ocular Drug Delivery

Ocular inserts are mostly sterile drug-impregnated solid preparations with multiple layers, usually containing a drug reservoir and an annual ring protected inside a rate-controlling membrane. They are classified based on their physicochemical properties, which include soluble and insoluble ocular inserts. Soluble or erodible ocular inserts undergo gradual dissolution while releasing the drug, so manual removal is not needed. On the other hand, insoluble ocular inserts are able to deliver drugs at a controlled, predetermined rate, but manual removal is needed. They are inserted into the conjunctival sac of the eye for the main purpose of prolonging the drug retention time on the surface [102].

Langston et al. developed the first ocular insert drug delivery system for the management of herpes keratitis. Idoxuridine was the active ingredient of this formulation. In general, the condition of the infected rabbit eyes was more improved with treatment with the idoxuridine ocular insert, eye drop, and ointment formulation than in the control group. In comparison, the 100 µg/h ocular insert showed the most improvement in the rabbit eyes in comparison to 0.5 µg/h, 7 µg/h, 15 µg/h, and 30 µg/h ocular inserts, 0.1% eye drops, and a 1% ointment formulation. Besides that, improvement could be seen within 24 h after the application of ocular inserts, but only after 92 h of application with the idoxuridine eye drops and ointment. Moreover, the eye infection continued to worsen clinically during the first 24 h of therapy in the idoxuridine eye drops and ointment group. Thus, ocular inserts have an outstanding in vivo efficacy compared to eye drops and ointment formulations [103].

## 5. Clinical and Safety Aspect of Novel Ocular Delivery Approaches

The safety profile is important to take into consideration in order to develop safe and efficient ophthalmic formulations. The purpose of developing novel drug delivery systems is to lengthen the retention time of therapeutic agents on ocular surfaces. The prolonged direct contact of the formulation with the ocular tissue may cause toxicity or irritation.

Currently, there are no ongoing clinical trials on novel drug delivery for ocular HSK. Previously, Sirion Therapeutics Inc. formulated a pH-responsive in situ ophthalmic gel containing ganciclovir 0.15% as the main active ingredient targeting HSK. The formulation was approved by the FDA in 2009 with the trademark Zirgan^®^ [104]. Ganciclovir was formulated with carbomer 974P, water for injection, sodium hydroxide, mannitol, and a proper amount of benzalkonium chloride [104]. There was a total of four clinical trials conducted. Firstly, comparing the healing rate of acyclovir 3% ophthalmic ointment with ganciclovir 0.15% gel in a sample size of 164 with HSK condition in one open-label, randomised, controlled, and multicentre trial revealed no substantial difference. Another three clinical trials with the same purpose carried out in a sample size of 213 in single-blinded, randomised, controlled, and multicentre trials also revealed no difference in healing rate [104,105]. The optimal dose in these trials was five times daily with one drop instillation until fully healed, followed by three times daily for a week. During the trials, the adverse complaints reported included blurred vision, ocular irritation, punctate keratitis, and conjunctival hyperaemia, but were all reported to be low [9,105]. Post-marketing surveillance revealed that occasional eye irritation symptoms, such as mild burning, a tingling sensation, or blurred vision, were reported to be rare and did not mandate it to be withdrawn from the market. Furthermore, the ocular in situ gel formulation was also proven to improve clinical effectiveness for patients with HSK. For instance, a multicentre, randomised, investigator-masked, parallel group-controlled trial carried out by Lin et al. showed that the total clinical effectiveness was 95.10% for the ganciclovir in situ ocular gel group and 93.00% for the ganciclovir ocular gel group. These outcomes implied that the 0.15% GCV in situ ophthalmic gel is more effective for the management of HSK than the 0.15% GCV conventional ocular gel. Moreover, less blurred vision upon administration was experienced by patients using the in situ ocular gel for their treatment of herpes keratitis. A controlled trial proved that discomfort, blurred vision, or difficulty of dividing doses upon administration were encountered by patients in the conventional ocular gel group. The maximum percentage of patients that complained about blurred vision upon administration was 27.03% in the ganciclovir conventional ocular gel group, while in the ganciclovir in situ ocular gel group, it was only 12.96%. Thus, it can be seen that the in situ ocular gel caused less blurred vision upon administration than non-in situ ocular gel [97].

Alternatively, another study is recruiting patients for the injection of genetic material into the cornea of infected patients. This gene editing therapy introduces BD111 CRISPR/Cas9 mRNA for the treatment of refractory herpetic viral keratitis in order to prevent infectious blindness in those patients. It aims to obtain the dose related toxicities, maximum tolerated dose, effectiveness, and recurrence possibility of the same infection [106]. Therefore, we could conclude here that the progress of the pharmaceutical formulation approach in the treatment of this ocular viral infection is largely confined within the laboratory barrier, and we hope to receive promising reports to move into human trials in the near future.

## 6. Conclusions

The eye is the primary sensory organ for vision, acting as the window to the world. Different structures within the eyes must function closely in harmony with one another, allowing humans to capture nature’s beauty and build connections with their surroundings. However, as the eyes are exposed directly to the external environment, they are susceptible to the vicissitudes of diseases. Herpes virus infection in the eyes can appear asymptomatic for some people; but can be severe and catastrophic for others, especially those with weakened immunity. Therefore, prompt treatment is typically given to all patients to relieve symptoms and prevent worsening of the condition. Topical antiviral treatment remains the preferred choice owing to the ease of administration, less invasiveness, and affordable cost. Unfortunately, the well-established ocular self-defense mechanism limits the clinical effectiveness of the current topical antiviral formulations. For instance, the blinking reflex and tear turnover give rise to rapid precorneal drug elimination into the nasolacrimal duct, causing undesirable systemic absorption and meagre ocular bioavailability. Ointments are better in prolonging precorneal contact time, but many reported blurred vision and difficulty in administration, leading to poor patient acceptance and compliance. Regardless of the challenges in ophthalmic preparations, numerous novel approaches have been developed to obviate the obstacles faced by conventional ocular formulations over the years. Undeniably, the development of in situ gel-forming solutions is one of the best, most applaudable research outcomes. In fact, it was found that the novel formulation provides good adherence characteristics with lower polymer concentration, hence the minimal risk of toxicity compared to conventional formulations. Such smart ocular delivery, in essence, boosts the therapeutic outcome by prolonging the drug release and contact time attributed to the sol–gel transformation and mucoadhesive behaviour. In addition, the amalgamation of the nanotechnologies, prodrugs, and peptide drug delivery with in situ gelling systems boosts the ocular targeting efficiency. Most of the present studies showed that stimulus-responsive in situ formulations are generally safe, while a minority reported mild irritation, which can be modified to improve ocular tolerance. Therefore, this current review concludes that the novel drug delivery approach is one of the preferred drug delivery systems for treating ocular herpetic infection. Nevertheless, the majority of the studies were conducted preclinically with animal models within a short observation duration. Indubitably, future additional clinical investigations are mandatory to evaluate the reproducibility, safety, and toxicity of the advanced topical formulations to avoid regulatory hurdles during commercialisation.

## Figures and Tables

**Table 1:** Lipid-based nanocarriers for ocular delivery against herpes simplex virus keratitis.

Objectives	Drug	Type of Formulation	Polymer Used	Membrane/Cell Line/Animal Model	Outcome	Source
To investigate the pharmacokinetics of acyclovir liposomes delivered to aqueous humour.	Acyclovir	Liposomes	Cholesterol, L-phosphatidylcholine, stearylamine	New Zealand (NZ) albino rabbits	Particle size: 370.9 ± 5.6 nm. Entrapment efficiency: 22.8%. Loading ACV concentration in liposome dispersion: 0.20 mg/mL. In vivo efficacy: 11-fold greater drug availability in the aqueous humour vs. reference ointment. In vitro release: higher drug release (50.25%).	[40]
To develop and optimise formulations of transferrin-conjugated liposomes containing ganciclovir.	Ganciclovir	Liposomes	Cholesterol, 1,2- distearoyl-*sn-*glycero-3-phosphocholine (DSPC), 1,2- distearoyl-*sn-*glycero-3-phosphoethanolamine-N-[amino(polyethylene glycol)-2000] (DSPE-PEG), 1,2-distearoyl-sn-glycero-3-phosphoethanolamine-N-[maleimide (polyethylene glycol) − 2000] (DSPE-PEG-MAL)	Human retinal pigment epithelial cells (ARPE-19)	Particle size: 88–113 nm. Zeta potential: ~−32 mV. Entrapment efficiency: 32–36%. In vitro release: prolonged drug release of over 12 h. In vivo efficacy: higher drug uptake by ARPE-19. In vitro cytotoxic (APRE-19): cell viability of 80–100% based on MTT assay.	[42]
To evaluate the ocular retention and intraocular delivery of mucoadhesive niosomal ganciclovir.	Ganciclovir	Niosomes coated with chitosan	Cholesterol, Span 60, chitosan	NZ albino rabbits	Particle size: 190 nm. Zeta potential: +41.8 mV (successfully coated with cationic chitosan). Entrapment efficiency: 47.2%. In vitro release: sustained drug release over 12 h. In vivo efficacy: drug concentrations obtained in the aqueous humour of niosome-treated albino rabbits were significantly greater. In vivo irritation: no visual irritation or damaging effect to ocular tissues of tested rabbits.	[43]
To fabricate and achieve efficient delivery of valacyclovir into the eye via solid lipid nanoparticles (SLNs).	Valacyclovir	SLNs	Stearic acid, tristearin, poloxamer 188, sodium taurocholate	Chorioallantoic membrane (CAM)	Particle size: 202.5 ± 2.56 nm. Zeta potential: −34.4 ± 3.04 mV. Entrapment efficiency: 58.82 ± 2.45%. In vitro release: sustained drug release over 12 h. In vivo efficacy: improved ocular bioavailability. Ex vivo irritation: no irritation in CAM and histopathology result.	[45]
To improve the ocular bioavailability of acyclovir using SLN and nanostructured lipid carriers (NLC) delivery systems.	Acyclovir	SLNs and NLCs	Stearic acid, Capryol^®^ 90 Lauroglycol^®^ 90, Compritol^®^ 888 ATO, and Cithrol GMS, Tween^®^ 40, Tween^®^ 80, Poloxamer^®^ 188, Brij^®^ 78	Bovine cornea	Particle size: 185–766 nm. Zeta potential: −30 to 34 mV. Entrapment efficiency: 4–34%. In vitro release: Both NLCs and SLNs showed extended drug release (8 h) compared to the reference solution (4 h). Faster diffusion and release of drug from the NLCs. Hydration level: no signs of toxicity to the cornea based on hydration level test.	[46]
To conduct an ex vivo and in vivo evaluation of chitosan-coated NLCs for acyclovir ocular delivery.	Acyclovir	NLCs	Lauroglycol^®^ 90, Compritol^®^ 888 ATO, Cithrol GMS, Tween^®^ 40, chitosan	Vero cells	In vivo efficacy: 3.5-fold reduction in effective concentration to achieve 50% inhibition of viral replication (IC_50_) was observed with acyclovir NLC-treated monkey kidney cells (CV-1). Acyclovir uptake by primary human corneal epithelial cells (HCEC) was higher in NLCs. In vitro cytotoxicity: MTT assay found no toxic effects on Vero cells.	[47]
To develop and characterise a nanoemulsion of acyclovir as a topical gel.	Acyclovir	Nanoemulsion	Castor oil, Span 40, PEG 400	-	Mean vesicle size: 41.6 nm. Zeta potential: −32.4 mV. Loading capacity: ~62–89%. In vitro release: 88% drug release within 8 h.	[50]

**Table 2:** Polymeric-based nanocarriers for ocular drug delivery against herpes simplex virus keratitis.

Objectives	Drug	Type of Formulation	Polymer Used	Membrane/Cell Line/Animal Model	Outcome	Source
To evaluate the solubility of acyclovir, corneal permeability, and sclera penetration of Soluplus and Solutol polymeric micelles.	Acyclovir	Polymeric micelle	Soluplus^®^ (polyvinyl co- prolactam-polyvinyl acetate-polyethylene glycol copolymer)	Chorioallantoic membrane (CAM)	Particle size: 219 nm. Zeta potential: +0.35 mV. Encapsulated acyclovir solubility: 2-fold in both water medium and phosphate-buffered saline (PBS) compared to unencapsulated acyclovir. In vivo permeability: 2.8-fold and 3.4-fold higher permeability flux than aqueous acyclovir in both cornea and sclera. In vitro irritation: no toxicity in fertilised eggs.	[54]
To develop a clear aqueous nanomicelle formulation and evaluate its biocompatibility.	Biotinylated lipid prodrug of acyclovir	Surfactant nanomicelle	D-α-tocopheryl polyethylene glycol 1000 succinate (Vitamin E TPGS) and octoxynol-40	Human corneal epithelial cells (HCECs)	Particle size: 10.78 nm. Zeta potential: −1.59 mV. Entrapment efficiency: ~90%. In vitro release: showed sustained release properties for up to 4 days. In vitro ocular irritation: no cytotoxic effect in HCECs.	[58]
To validate the effect of acyclovir concentration on the physicochemical characteristic and release profile of chitosan nanoparticles.	Acyclovir	Polymeric nanosuspension	Chitosan and Tween-80	-	Particle size: 200 ± 30 nm. Zeta potential: +36.7 ± 1.5 mV. Encapsulation efficiency: 56%. Loading capacity: 25%. In vitro release: release for up to 24 h.	[64]
To validate the effect of chitosan concentration on the physicochemical characteristic and release profile of chitosan nanoparticles.	Acyclovir	Polymeric nanosuspension	Chitosan and Tween-80	-	Particle size: 250 nm. Zeta potential: +42.8 mV. Encapsulation efficiency: 90%. Loading capacity: 50%. In vitro release: release for up to 24 h.	[65]
To increase ocular bioavailability of acyclovir through poly (lactic-co-glycolic acid) (PLGA)-based nanoparticles stabilised with vitamin E TPGS.	Acyclovir	Polymeric nanosuspension	PLGA and vitamin E TPGS	Albino rabbits	Particle size: 262.38 ± 11.85 nm. Zeta potential: 15.14 ± 2.81 mV. Encapsulation efficiency: 74.12 ± 6.19%. Loading capacity: 8.65 ± 1.09%. In vitro release: showed sustained release for up to 72 h. In vivo permeability: 1.4-fold higher permeability flux compared to drug solution. In vivo distribution: bioavailability was 2.76-fold higher than drug solution. In vivo ocular irritation: demonstrated mild irritation but subsided after 6 h.	[66]

**Table 3:** Prodrug approach for ocular drug delivery against herpes keratitis.

Objectives	Drug	Type of Formulation	Polymer Used	Membrane/Cell Line/Animal Model	Outcome	Source
To evaluate the corneal absorption of amino acid prodrugs.	Acyclovir (ACV)	Ophthalmic prodrug	-	Primary corneal epithelial cell cultures	Stability: L-Serine-ACV (SACV) was the most stable among the other prodrugs. In vivo ocular absorption: SACV and L-Valine- ACV (VACV) showed a 2-fold increase in area under concentration time curve (AUC) and maximum aqueous humor concentration (C_max_) of prodrug and regenerated ACV compared to ACV. Cytotoxicity studies: cellular toxicity of ACV prodrugs was significant lower compared to trifluridine.	[73]
To characterise the amino acid prodrugs based on affinity and permeability.	Acyclovir	Ophthalmic prodrug	-	Rabbit primary corneal epithelial cell culture (rPCEC)	In vitro antiviral studies: SACV displayed anti-HSV-1 activity and the concentration required to inhibit viral cytopathogenicity by 50% (EC_50_) was 6.3 μM. Corneal permeability: SACV exhibited higher corneal permeability and superior anti-HSV-1 activity relative to ACV.	[74]
To evaluate dipeptide monoester ganciclovir (GCV) prodrugs.	Ganciclovir (GCV)	Ophthalmic prodrug	-	New Zealand White (NZW) rabbits	Solubility: the prodrugs showed better aqueous solubility compared to parent drug. Transcorneal permeability: valine-GCV (VGCV) and divaline-GCV (VVGCV) were 7- to 8-fold higher than GCV. In vivo efficacy studies: 1% VVGCV has better therapeutic activity against HSV-1 epithelial keratitis compared to 1% trifluridine.	[75]
To evaluate the corneal absorption of dipeptide monoester prodrugs.	Ganciclovir	Ophthalmic prodrug	-	NZW rabbits	In vivo studies: The area under the concentration–time profile (AUC_infinity_)of the regenerated GCV from tyrosine-valine-GCV (YVGCV) was 8.6-fold higher than GCV, whereas VVGCV was 1.8-fold higher than GCV. Both YVGCV and VVGCV demonstrated enhanced permeability and superior corneal absorption.	[76]
To develop sodium-dependent multivitamin transporter (SMVT)-targeted biotinylated lipid prodrugs to improve cellular absorption.	Acyclovir	Ophthalmic prodrug	-	Human corneal epithelial cells (HCECs)	Uptake study: the uptake of biotin-ricinoleicacid-acyclovir (B-R-ACV) and biotin-12hydroxystearicacid-acyclovir (B-12HS-ACV) was nearly 13.6-fold and 13.1-fold higher than parent drug, respectively. Stability: B-R-ACV and B-12HS-ACV possessed better stability. In vitro antiviral activity: B-R-ACV: ~4.5-fold and 8.7-fold more potent against HSV-1 and HSV-2, respectively, compared to parent drug. B-12HS-ACV: ~200-fold and 21-fold more potent against HSV-1 and HSV-2, respectively, compared to parent drug.	[77]

**Table 4:** Approaches to ocular delivery of peptides against herpes simplex virus keratitis.

Objectives	Drug/Peptide Used	Type of Formulation	Polymer Used	Membrane/Cell Line/Animal Model	Outcome	Source
To evaluate antimicrobial activity of LL-37.	LL-37	Ophthalmic peptide delivery	-	Human corneal and conjunctival epithelial cells (LL-37 expression study)	Antiviral assay: 500 µg/mL of LL-37 reduced HSV-1 viral load by more than 100-fold compared to the phosphate-buffered saline (PBS) and scrambled peptide.	[82]
To compare the release of LL-37 from nanoparticle–hydrogel corneal implants and human corneal epithelial cell (HCEC)-produced LL-37.	LL-37	Peptide delivery, nanoparticle–hydrogel corneal implants, human corneal epithelial cell (HCEC)-produced LL-37.	-	HCECs	In vitro studies: the viral binding was reduced by LL-37, but the virus was not completely cleared from the already infected cells.	[83]
To identify peptides that bind specifically to heparan sulfate (HS). To investigate their effectiveness in inhibiting HSV-1.	G1 and G2 peptide	Ophthalmic peptide delivery	-	Mouse cornea	In vivo studies: the G1 and G2 peptides significantly reduced the severity of keratitis when administered prophylactically.	[85]
To develop and evaluate G2-C contact lens to lengthen the release of G2-C peptide.	G2-C peptide	Ophthalmic peptide delivery using contact lens.	-	Human cornea epithelial cells (ex vivo virus spread assay), pig corneas (ex vivo study of inhibition of HSV-1), mouse model (in vivo efficacy study)	In vitro release: the release of G2-C was prolonged with the use of the contact lens. In vivo and ex vivo studies: the G2-C lens were effective in inhibiting HSV-1 entry in both ex vivo and in vivo studies.	[87]
To evaluate of the therapeutic efficacy of 1% apoEdp.	apoEdp	Ophthalmic peptide delivery	-	Mouse	In vivo studies: 1% apoEdp was as effective as 1% trifluridine in reducing the incidence and severity of herpes simplex keratitis (HSK). The expression of several proinflammatory cytokines was downregulated compared to the control.	[88]
To evaluate the efficacy of 1% apoEdp against HSV-1 thymidine kinase (TK)-positive and HSV-1 TK negative virus.	apoEdp	Ophthalmic peptide delivery	-	NZW rabbits	In vivo studies: apoEdp was as effective as trifluridine and foscarnet in reducing the severity of keratitis in both TK-positive and TK-negative HSV groups.	[89]
To develop ocular insert for antimicrobial peptide delivery.	hLF 1-11	Ophthalmic peptide delivery		-	hLF 1-11 was found to be stable in a freeze-dried solid matrix of hydroxypropyl methylcellulose (HPMCs) and it released the peptide in sustained manner.	[90]

**Table 5:** Smart ocular delivery using in situ gel approach against ocular diseases.

Objective	Drug	Types of Stimuli/Polymer Used	Membrane/Cell Line/Animal Model	Outcome	Source
Preparation of ocular in situ micelles to enhance ocular permeation.	Acyclovir	Thermo-responsive micelles/Soluplus	Rabbits	Higher corneal and sclera permeability compared to conventional formulation.	[54]
Preparation and evaluation of ion-activated in situ gel ophthalmic delivery system of acyclovir based on kappa-carrageenan.	Acyclovir- hydroxypropyl-β-cyclodextrin complex	Ion-activated/kappa-carrageenan	New Zealand White (NZW) rabbits	Rheology: pseudoplastic fluid Gelling capacity: gel formed rapidly after contact with tear fluid, maintained for a long time. In vitro release: 80% of drug released after 6 h In vitro permeability: 2.16-fold higher apparent permeability. In vivo irritation: no irritation to rabbits’ eyes.	[95]
To develop sustained release nanoparticles loaded with ganciclovir prodrug.	Ganciclovir prodrug	Thermo-responsive/poly (lactic-co-glycolic acid) (PLGA), PLGA-polyethylene glycol-PLGA (PLGA-PEG-PLGA)	Human corneal epithelial cells (HCECs)	NPs were small in size with higher drug loading and entrapment. Biphasic release pattern: burst release followed by sustained release. Efficient permeation of prodrug with accumulation in cul-de-sac.	[93]
To develop and evaluate thermo-responsive in situ gel nanoemulsions in delivering acyclovir.	Acyclovir (ACV)	Thermo-responsive nanoemulsion/Triacetin and Transcutol^®^ P (nanoemulsion) poloxamer 407 and poloxamer 188 (in situ)	NZW rabbits (in vivo ocular irritation test) and Hen’s Egg-Chorioallantoic Membrane (HET-CAM) (in vitro ocular irritation test)	Gelation temperature 30.9 °C. pH: 4.58 ± 0.068 Viscosity: 103.03 ± 4.68 mPa.s In vitro drug release efficiency: 80.78 ± 1.82% The optimised formulations displayed sustained release. Ex vivo permeation: permeation of ACV was 2.83-fold higher in optimised formulation compared to ACV solution. In vivo ocular irritation test: minimal conjunctival redness but disappeared after 2 h of administration. In vitro ocular irritation (HET-CAM) test: cumulative score of 0.33 ± 0.58, indicating non-irritant	[94]
To design polymeric nanoparticles of acyclovir incorporated in in situ gelling system to provide dual sustained release effect, whereby the duration of action and bioavailability through different routes of administration could be improved.	Acyclovir	Thermo-activated/Pluronic F-127 and pH-activated/Carbopol	-	Gelation temperature: 25 ± 0.20 to 35 ± 0.46 °C Gelation time: 2 to 4 min In vitro drug release study: better sustained release characteristics, with non-Fickian diffusion mechanism of drug release.	[96]
Formulation of valcyclovir in situ gels.	Valcyclovir	pH-activated/Carbopol 940, HPMC K 100M	-	In situ gels show sustained release with profile.	[98]
Development of acyclovir-loaded niosomes entrapped in hydrogel	Acyclovir	pH-activated/Span 20 or Span 60, cholesterol, Carbopol 934, methylcellulose	Rabbits	Sustained release with no sign of irritation	[99]

## Data Availability

Data is freely available.
